# Changing epidemiology of lower extremity fractures in adults over a 15-year period – a National Hospital Discharge Registry study

**DOI:** 10.1186/s12891-021-04291-9

**Published:** 2021-05-19

**Authors:** Philipp Hemmann, Maximilian Friederich, Daniel Körner, Tim Klopfer, Christian Bahrs

**Affiliations:** 1 Department of Traumatology and Reconstructive Surgery, BG Trauma Centre Tuebingen, Eberhard Karls University Tuebingen, Schnarrenbergstrasse 95, 72076 Tuebingen, Germany; 2 Eberhard Karls University Tuebingen, Medical School, Geissweg 5, 72076 Tuebingen, Germany; 3 Orthopädische Chirurgie Bayreuth, Parsifalstraße 5, 95445 Bayreuth, Germany; 4 Department of Orthopaedics and Trauma Surgery, Schön Klinik Neustadt, Am Kiebitzberg 10, 23730 Neustadt in Holstein, Germany

**Keywords:** Fracture, Lower extremity, Incidence, Epidemiology, Geriatric

## Abstract

**Background:**

Demographic changes led to an increasingly ageing population in Germany and thus to possible changes in the frequency of fractures. The primary aim of this study was to report changes in fracture rates of the lower extremities in Germany in 2002 compared to 2017 and to evaluate those changes.

**Methods:**

Inpatient data from the German National Hospital Discharge Registry (ICD10) for 2002 and 2017 were evaluated. Changes in total counts and incidence rates were analysed for fractures in the following locations: femoral neck, pertrochanteric, subtrochanteric, distal femur, femoral shaft, proximal and distal tibia, tibial shaft, medial and lateral malleolus, and other parts of the lower leg (including bi- and trimalleolar fractures), calcaneus, talus, other tarsal bones, metatarsal bones, greater toe, lesser toe, other fractures of foot or unspecific fractures of foot and toe. Patients were classed into age groups by sex: 15–24, 25–34,35–44, 45–54, 55–64, 65–74, 75–84, 85–90 and >  90 years.

**Results:**

The total count for lower extremity fractures in men and women increased slightly by 4.5% from 305,764 in 2002 to 319,422 in 2017. Hip and femur fractures increased by 23.5% from 150,565 in 2002 to 185,979 in 2017. The number of these fractures among men increased by 46% and among women by 15.3%. The total count of lower leg fractures decreased by 15.4% from 131,162 in 2002 to 110,924 in 2017. Especially, younger age groups showed a decline for all tibial segments and ankle fractures. For both sexes, the number of lower leg fractures in those 75 years or older increased in all lower leg fracture locations. Most femur and lower leg fractures occurred in women. The incidence of fractures rose sharply from 2002 to 2017, especially for older cohorts.

**Conclusion:**

The total numbers of lower extremity fractures increased slightly in 2017 compared to 2002 – especially hip and femur fractures among men. The incidence of almost all lower extremity fracture types among older people increased during this time. Women were particularly affected. Therefore, focused prevention programmes should be considered including an extended fracture spectrum in the elderly.

## Introduction

Demographic change is leading to an increasingly ageing population in Germany. Between 1990 and 2018 the number of people over the age of 67 years increased by 54%. According to the German Federal Office of Statistics, the population of older age groups will increase by another 5 to 6 million by 2039, reaching at least 21 million [1]. A corresponding increase in geriatric fractures can also be expected, which means high costs for the healthcare system [2]. Aigner et al. reported that hip fractures costs 8853 € in a German University Hospital while reimbursement was 8196 € [3] making a deficit of 657 €. Fractures in the elderly will pose challenges for attending physicians in particular, as they will be confronted with an increasingly geriatric and multimorbid patient population. The main causes of these fractures are low-energy traumas (e.g. falls from a standing position) in combination with osteopenia. It has been shown that the highest absolute number of fractures occur in individuals with osteopenia [4]. Osteoporosis is highly prevalent among people in the fifth decade of life or older [5, 6]. Women are up to five times more likely to develop osteoporosis than men [7] and suffer about two-thirds of all osteoporotic fractures worldwide [8]. Fragility fractures are mainly located in the proximal humerus, distal radius, proximal femur, pelvis and spine [9–12].

The literature includes high-quality register studies, mainly from Scandinavia, which evaluate changes in frequencies of different fractures over time [2, 13–20]. However, there are only a few epidemiological studies on changes over time in the frequency of geriatric fractures in Germany. These studies have particularly focused on hip fractures [12, 21–23]. Fractures of the upper extremity have been investigated by the authors in a previous study [21] which is why a further study on the frequency of fractures in the lower extremity seems useful. To the best of our knowledge, there is no recent study explicitly on the epidemiology of lower extremity fractures in Germany and the associated investigation of fracture frequencies and their distribution.

The goal of the present study was to analyse the epidemiological development of fractures of the lower extremities from 2002 to 2017 in Germany. Fractures were evaluated for persons ≥15 years of age, with a focus on older people, by analysis of the national hospital discharge diagnosis register. In addition, the study presents changes in total counts and incidence rates for various lower extremity fractures.

## Patients and methods

Data were retrieved from the national hospital discharge diagnosis register (www.gbe-bund.de) from 01/01/2002 to 31/12/2002 and from 01/01/2017 to 31/12/2017. This database is maintained by the Robert Koch Institute and the German Federal Office of Statistics and is responsible for recording data on all inpatients treated in Germany. The register covers over 99% of all German hospitals and thus is a source of accurate epidemiological data. The online database (GBE) brings together health data and health information from more than 100 different sources in a central location, including many surveys of the statistical offices of the Federation and the states, but also surveys of numerous other institutions from the health sector. GBE’s thematic fields include all areas of the health care system: General conditions of the health care system, health situation, health behaviour and health risks, health problems and diseases, health care, health expenditure, costs and financing of the health care system.

The level of trauma (e.g., Injury severity Score, Anderson and Gustillo classification, AO/OTA-classification) is unfortunately not provided by the register. The register only counts inpatient cases and therefore only contains the ICD-10-code. The register only uses the first three digits of the ICD-10 code. Therefore, the register could classify fractures only according to bone and bone segment. The Robert Koch Institute and the German Federal Office of Statistics are responsible for recording data on all inpatients treated in German hospitals. Therefore, it provides accurate and reliable data and fractures with specified codes were obtained and included. Exclusion criteria were not applied.

Data were selected for retrieval based on the International Classification of Diseases (ICD-10-GM: 10th Revision, German Modification) [24]. The ICD-10-GM was introduced in 2000 as the successor to the ICD-9. To avoid documentation errors and incorrect coding, the year 2002 was chosen as the starting point for study analysis.

The following fracture locations were chosen.

Hip and femur: femoral neck S72.0; pertrochanteric femur S72.1; subtrochanteric femur S72.2; femoral shaft S72.3; distal femur S72.4; multiple fractures of the femur S72.7; other parts of the femur S72.8; and femur, part unspecified S72.9. For assessing changes in total counts and ratios, per- and subtrochanteric femoral fractures (S72.1, S72.2) were grouped together. Additionally, multiple fractures of the femur (S72.7); other parts of the femur (S72.8); and femur, part unspecified (S72.9) were grouped as ‘other parts’.

Lower leg: proximal end of the tibia S82.1, tibial shaft S82.2, distal end of the tibia S82.3, medial malleolus S82.5, lateral malleolus S82.6 and other parts of the lower leg (including tri- and bimalleolar fractures) S82.8.

Foot: calcaneus (S92.0), talus (S92.1), other and unspecific tarsal bone(s) (S92.2), metatarsal bone (S92.3), great toe and other toes (S92.4, S92.5) as well as other parts (S92.7, S92.9) were grouped together.

Men and women were divided by sex into nine age groups: 15–24, 25–34,35–44, 45–54, 55–64, 65–74, 75–84, 85–90 and >  90 years.

The authors wanted to show the fracture development in all age groups but children. Previous studies by Körner et al. examined fracture patterns in children for the upper and lower extremity [25, 26]. Therefore, age groups < 15 years were not considered. Although, geriatric fractures are increasing, the development of fracture frequencies and incidences in the younger and working age groups are also important. Furthermore, the data query from the register provides the age ranges. A grouping with more than 10 years within a group seemed inaccurate to the authors.

The register provides total counts for the selected fractures as well as incidence rates. The percentage changes in total numbers between 2002 and 2017 were also calculated. The incidence rate ratio (IRR) was calculated by dividing the 2017 incidence by the 2002 incidence.

Furthermore, the composition of the German population by age group and gender in 2002 and 2017 were queried.

Statistical analysis was performed using Microsoft Office Excel 365 ProPlus (Microsoft Corporation, Redmond, USA).

The study was conducted in agreement with the ethical standards of the institutional and national research committee and with the 1964 Helsinki declaration and its later amendments. This is an epidemiological study with anonymized, centrally collected, and online publicly available data. No patient consent or approval of the local ethics committee was required.

## Results

The German population changed over the last 15 years. The percentage of older people grew. This can be first seen in the age group 45–54 years and all the following age groups (see Table 1). A more detailed view on the average change of every age group gives Table 1.

**Table 1:** Population of each age group for 2002 and 2017

Age group		2002	2017	Δ(%)
15–24 years	All sexes	9.514.459	8.683.081	−8,7
	Male	4.857.559	4.544.388	−6,4
	Female	4.656.900	4.138.693	−11,1
25–34 years	All sexes	10.751.372	10.588.332	−1,5
	Male	5.499.663	5.466.519	−0,6
	Female	5.251.709	5.121.813	−2,5
35–44 years	All sexes	14.012.379	9.951.567	−29,0
	Male	7.189.780	5.024.019	−30,1
	Female	6.822.599	4.927.548	−27,8
45–54 years	All sexes	11.301.177	12.911.332	14,2
	Male	5.693.657	6.517.216	14,5
	Female	5.607.520	6.394.116	14,0
55–64 years	All sexes	10.102.894	11.776.569	16,6
	Male	4.994.183	5.824.284	16,6
	Female	5.108.711	5.952.285	16,5
65–74 years	All sexes	8.217.435	8.323.603	1,3
	Male	3.793.315	3.936.643	3,8
	Female	4.424.120	4.386.960	−0,8
75–84 years	All sexes	4.769.704	7.120.634	49,3
	Male	1.597.063	3.071.104	92,3
	Female	3.172.641	4.049.530	27,6
85–90 years	All sexes	873.164	1.495.440	71,3
	Male	217.293	527.220	142,6
	Female	655.871	968.220	47,6
> 90 years	All sexes	578.516	770.034	33,1
	Male	130.577	191.277	46,5
	Female	447.939	578.757	29,2

A total of 305,764 fractures of the lower extremity were recorded in 2002. In 2017, there was an increase by 4.5% to 319,422 fractures. In 2002, women represented the majority with 61.1% (men: 38.9%). In 2017, the proportion of female fractures increased slightly to 62.3% (men: 37.7%).

A total of 150,565 fractures of hip and femur were registered in 2002. In 2017 there was an increase to 185,979 such fractures (23.5% increase). Women accounted for the majority of fractures, with a total count of 110,166 fractures (73.2%) in 2002 compared to men with 40,399 (26.8%). In 2017, the female proportion of all fractures decreased slightly to 68.3%, although the total number of fractures in women increased to 127,018. Fractures in men increased to 58,961 in 2017, representing 31,7% of all fractures.

Table 2 presents the total counts of hip and femur fractures for both sexes for the years 2002 and 2017 along with the percentage of change in these counts between the 2 years. For the three youngest male age groups, the counts decreased from 2002 to 2017 for every fracture type. For the age groups 55 years and older, total counts increased for every fracture type, with large percentage increases for men 75 years and older in fractures affecting the femoral neck, per- and subtrochanteric femur, femoral shaft and distal femur. In women, fracture counts decreased also in the youngest three age groups. With minor exceptions, total counts also increased for men starting at the age of 55 years and older.

**Table 2:** Total count, differences between 2017 and 2002 in percent for hip and femur fractures for men and women. Total count for patients > 90 years have been added together

	15–24 years			25–34 years			35–44 years			45–54 years			55–64 years			65–74 years			75–84 years			85–90 years			> 90 years		
	2002	2017	Δ (%)	2002	2017	Δ (%)	2002	2017	Δ (%)	2002	2017	Δ (%)	2002	2017	Δ (%)	2002	2017	Δ (%)	2002	2017	Δ (%)	2002	2017	Δ (%)	2002	2017	Δ (%)
***Male***																											
Femoral Neck (S72.0)	149	137	−8.1	246	176	−28.5	735	411	−44.1	1190	1452	22.0	2098	2590	23.5	3236	4042	24.9	4521	9408	108.1	2049	4479	118.6	1543	2841	84.1
Per- subtrochanteric (S72.1 + S72.2)	242	133	−45	360	277	−23.1	939	480	−48.9	1471	1203	−18.2	2183	2701	23.7	3470	4037	16.3	4374	8803	101.3	1705	4950	190.3	1283	3015	135.0
Femoral Shaft (S72.3)	1425	896	−37.1	643	519	−19.3	570	331	−42.0	355	446	25.6	337	553	64.1	396	596	50.5	270	1105	309.3	92	426	363.0	72	233	223.6
Distal Femur (S72.4)	548	331	−39.6	259	193	−25.5	341	193	−43.4	326	292	−10.4	349	397	13.8	294	318	8.2	173	399	130.6	41	142	246.3	28	69	146.4
Other parts (S72.7-S72.9)	448	24	−94.6	312	23	−92.6	328	19	−94.2	223	29	−87.0	194	37	−80.9	245	56	−77.1	216	115	−46.8	75	59	−21.3	45	25	−44.4
**Overall**	**2812**	**1521**	**−45.9**	**1820**	**1188**	**−34.7**	**2913**	**1434**	**−50.8**	**3565**	**3422**	**−4.0**	**5161**	**6278**	**21.6**	**7641**	**9049**	**18.4**	**9554**	**19,830**	**107.6**	**3962**	**10,056**	**153.8**	**2971**	**6183**	**108.1**
***Female***																											
Femoral Neck (S72.0)	47	28	−40.4	93	64	−31.2	367	170	−53.7	1029	1075	4.5	2701	3317	22.8	7526	6566	−12.8	19,631	20,482	4.3	10,548	11,221	6.4	8449	9305	10.1
Per- subtrochanteric (S72.1 + S72.2)	47	33	−29.8	84	65	−22.6	269	112	−58.4	646	577	−10.7	1569	2116	34.9	5892	5560	−5.6	18,830	20,076	6.6	10,885	15,702	44.3	9439	14,348	52.0
Femoral Shaft (S72.3)	373	307	−17.7	168	120	−28.6	172	101	−41.3	182	224	23.1	355	513	44.5	758	1050	38.5	1461	3121	113.6	658	1871	184.3	585	1525	160.7
Distal Femur (S72.4)	142	112	−21.1	112	74	−33.9	157	77	−51.0	207	270	30.4	457	608	33.0	877	909	3.6	1464	2103	43.6	639	1271	98.9	677	1212	79.0
Other parts (S72.7-S72.9)	146	9	−93.8	95	7	−92.6	109	11	−89.9	117	10	−91.5	213	38	−82.2	362	81	−77.6	861	286	−66.8	420	165	−60.7	347	126	−63.7
**Overall**	**755**	**489**	**−35.2**	**552**	**330**	**−40.2**	**1074**	**471**	**−56.1**	**2181**	**2156**	**−1.2**	**5295**	**6592**	**24.5**	**15,415**	**14,166**	**−8.1**	**42,247**	**46,068**	**9.0**	**23,150**	**30,230**	**30.6**	**19,497**	**26,516**	**36.0**

**Fig. 1:**
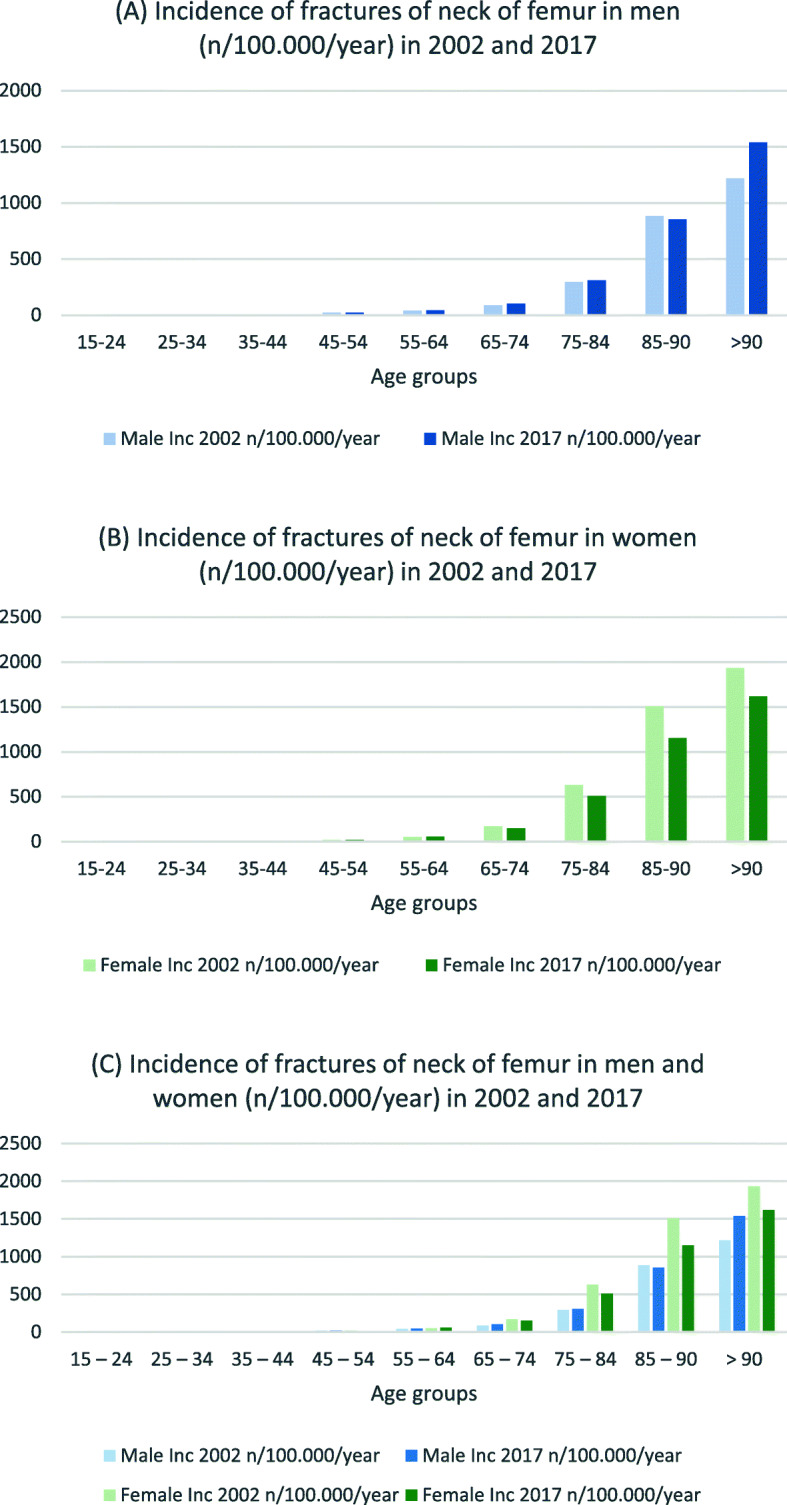
Incidences of proximal femoral neck fractures in **a** men, **b** women and **c** both (n/100,000/year) for 2002 and 2017

**Fig. 2:**
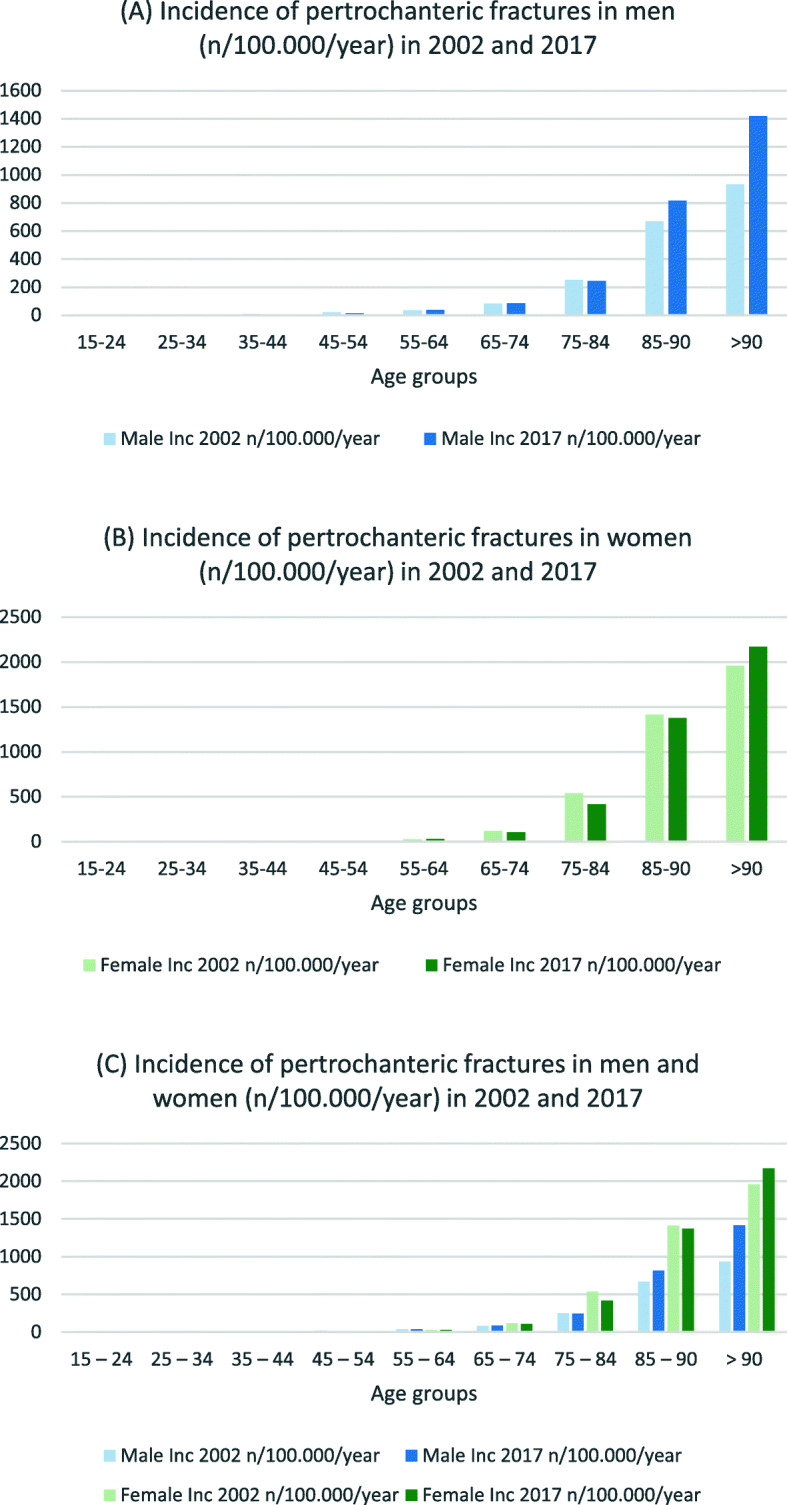
Incidences of pertrochanteric fractures in **a** men, **b** women and **c** both (n/100,000/year) for 2002 and 2017

**Fig. 3:**
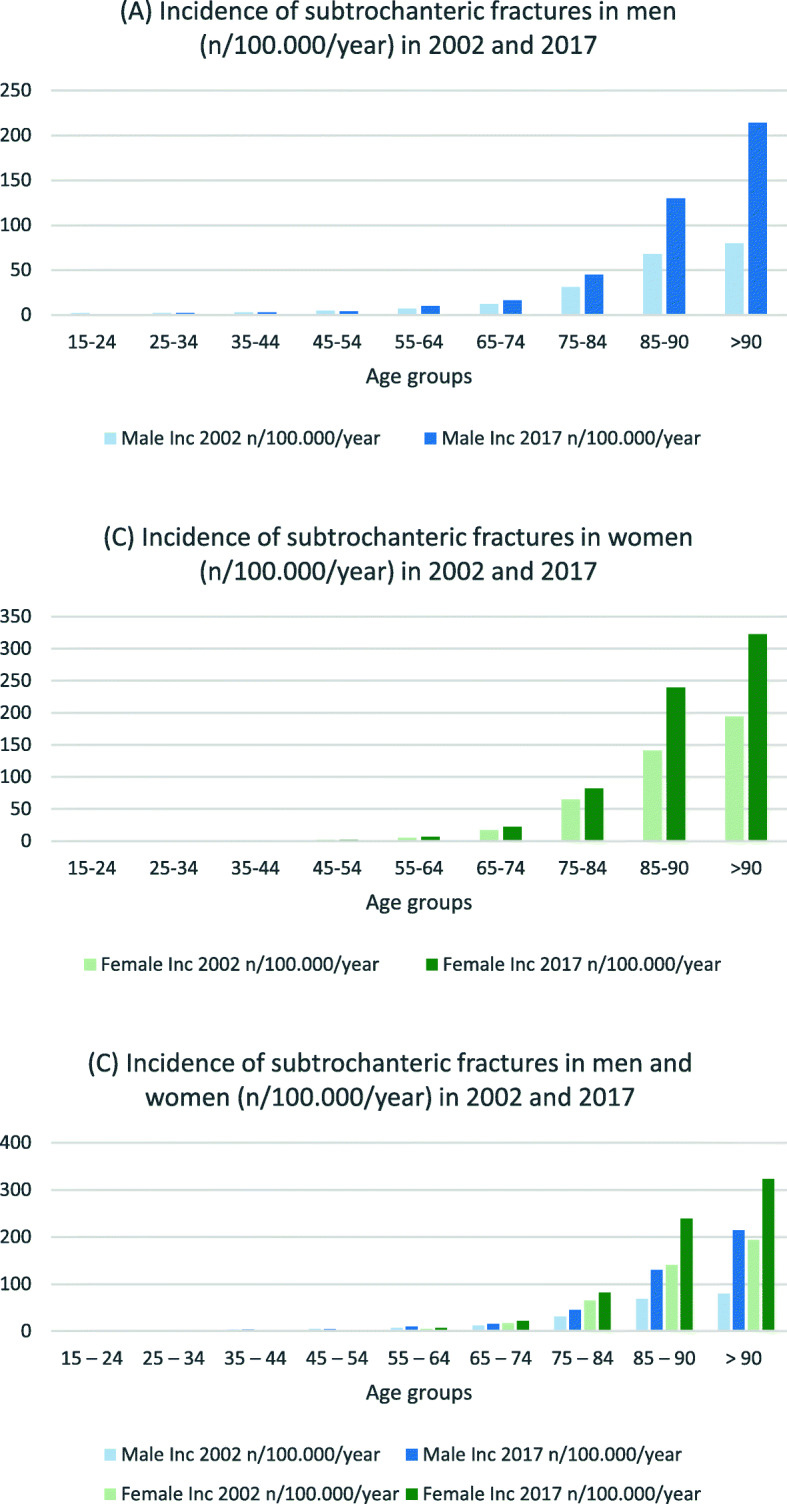
Incidences of proximal subtrochanteric fractures in **a** men, **b** women and **c** both (n/100,000/year) for 2002 and 2017

**Fig. 4:**
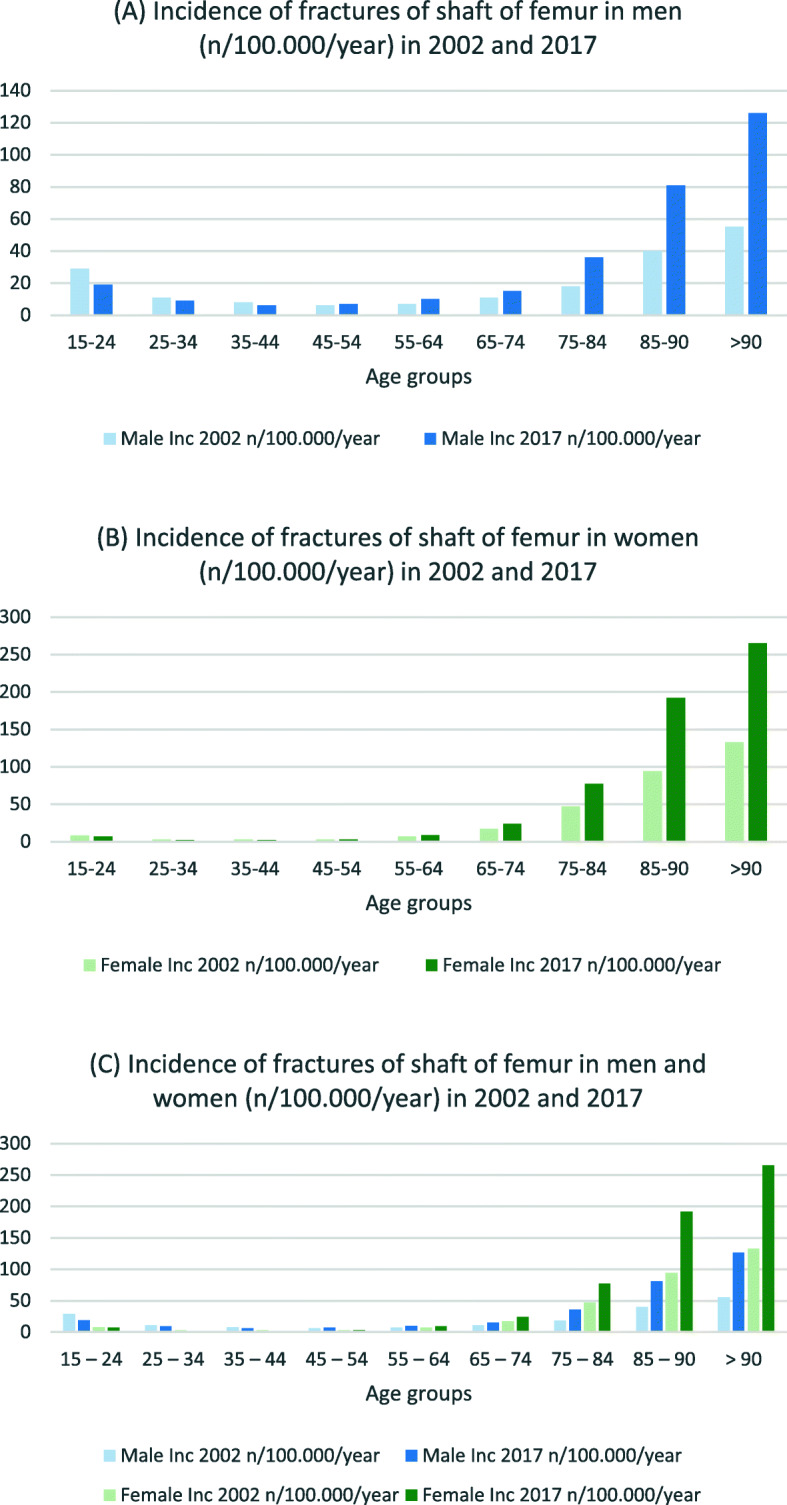
Incidences of femoral shaft fractures in **a** men, **b** women and **c** both (n/100,000/year) for 2002 and 2017

**Fig. 5:**
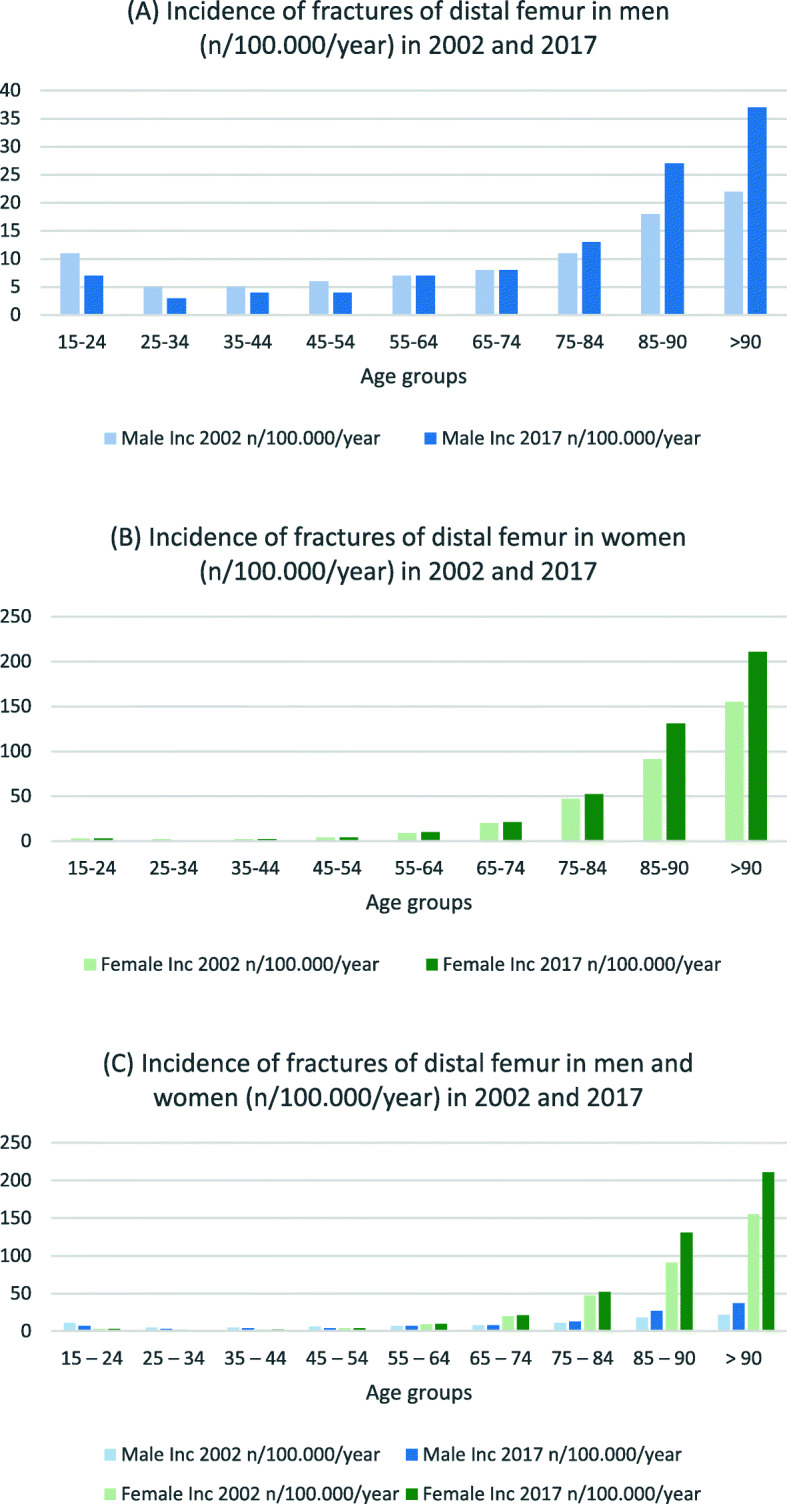
Incidences of distal femur fractures in **a** men, **b** women and **c** both (n/100,000/year) for 2002 and 2017

Table 3 presents incidence rates and IRR for hip and femur fractures in both sexes. For men in the three youngest age groups, the incidence for nearly all fracture types decreased (IRR 0.6–0.8) (see Figs. 1, 2 and 3). In the age groups 55 years and older, the incidence for nearly all proximal femoral fractures and femoral shaft fractures increased (IRR 1.1–2.7). The incidence and IRR of female femoral neck fractures decreased in nearly every group (0.5–0.9). Pertrochanteric fracture incidence rates also decreased in all groups except for women > 90 years (IRR 0.7–0.9). Subtrochanteric fracture incidences increased for all groups 55 years and older (IRR 1.3–1.4). The same could be seen for femoral shaft and distal femur fractures (IRR > 1.1) (see Figs. 4 and 5).

**Table 3:** Incidences of hip and femur fractures in 2002 and 2017 by gender (n/100,000/year), IRR: Incidence rate ratio

	15–24 years			25–34 years			35–44 years			45–54 years			55–64 years			65–74 years			75–84 years			85–90 years			> 90 years		
	2002	2017	IRR	2002	2017	IRR	2002	2017	IRR	2002	2017	IRR	2002	2017	IRR	2002	2017	IRR	2002	2017	IRR	2002	2017	IRR	2002	2017	IRR
***Male***																											
Femoral Neck (S72.0)	3	3	1.0	4	3	0.8	10	8	0.8	21	22	1.0	41	45	1.1	87	102	1.2	293	308	1.1	885	853	1.0	1217	1539	1.3
Pertrochanteric (S72.1)	3	2	0.7	4	3	0.8	10	7	0.7	21	14	0.7	36	37	1.0	81	86	1.1	252	243	1.0	669	815	1.2	931	1418	1.5
Subtrochanteric (S72.2)	2	1	0.5	2	2	1.0	3	3	1.0	5	4	0.8	7	10	1.4	12	16	1.3	31	45	1.5	68	130	1.9	80	214	2.7
Femoral shaft (S72.3)	29	19	0.7	11	9	0.8	8	6	0.8	6	7	1.2	7	10	1.4	11	15	1.4	18	36	2.0	40	81	2.0	55	126	2.3
Distal Femur (S72.4)	11	7	0.6	5	3	0.6	5	4	0.8	6	4	0.7	7	7	1.0	8	8	1.0	11	13	1.2	18	27	1.5	22	37	1.7
Multiple fractures of Femur (S72.7)	0	0	–	1	0	–	0	1	–	0	0	–	0	0	–	0	1	–	0	1	–	0	2	–	3	3	1.0
Other parts (S72.8)	0	0	–	0	0	–	0	0	–	0	0	–	0	0	–	0	1	–	1	1	1.0	4	4	1.0	2	2	1.0
Unspecified parts (S72.9)	9	0	0.0	5	0	0.0	0	4	–	0	3	–	0	3	–	1	5	5.0	3	12	4.0	7	26	3.7	9	28	3.1
***Female***																											
Femoral Neck (S72.0)	1	1	1.0	2	1	0.5	5	3	0.6	19	16	0.8	52	56	1.1	171	150	0.9	629	507	0.8	1506	1151	0.8	1929	1617	0.8
Pertrochanteric (S72.1)	1	1	1.0	1	1	1.0	3	2	0.7	10	7	0.7	26	29	1.1	117	105	0.9	538	415	0.8	1411	1373	1.0	1960	2168	1.1
Subtrochanteric (S72.2)	0	0	–	0	1	–	1	1	1.0	2	2	1.0	5	7	1.4	17	22	1.3	65	82	1.3	141	239	1.7	194	323	1.7
Femoral shaft (S72.3)	8	7	0.9	3	2	0.7	3	2	0.7	3	3	1.0	7	9	1.3	17	24	1.4	47	77	1.6	94	192	2.0	133	265	2.0
Distal Femur (S72.4)	3	3	1.0	2	1	0.5	2	2	1.0	4	4	1.0	9	10	1.1	20	21	1.1	47	52	1.1	91	131	1.4	155	211	1.4
Multiple fractures of Femur (S72.7)	0	0	–	0	0	–	0	0	–	0	0	–	0	1	–	0	1	–	1	2	2.0	2	4	2.0	2	4	2.0
Other parts (S72.8)	0	–	–	0	0	–	0	0	–	0	0	–	0	0	–	1	1	1.0	2	2	1.0	4	5	1.3	5	6	1.2
Unspecified parts (S72.9)	3	0	0.0	2	0	0.0	0	1	–	0	2	–	0	3	–	1	7	7.0	4	24	6.0	10	51	5.1	14	68	4.9

A total of 131,162 fractures of the lower leg were registered in 2002. In 2017 there was a decrease to 110,924 such fractures (− 15.4%). In 2002 women accounted for most lower leg fractures, with 68,231 (52%) cases compared to 62,931 for men (48%). In 2017, the proportion of these fractures that occurred in women rose to 56.1% despite a decrease in absolute numbers to 62,259 cases. For men, a decrease to 48,665 such fractures was documented, representing 43.9% of the total for 2017.

Table 4 shows total counts and percentage changes for lower leg fractures in both sexes for 2002 and 2017. For the two youngest male age groups lower leg fractures decreased for all locations. For men 75 years of age or older, counts increased for all lower leg fracture types. Similar to men, counts for all lower leg fracture types increased for women 75 years and older. It is noteworthy that although the total counts in women 75 years or older were much higher than those for men, the percentage increases from 2002 to 2017 were much greater in men.

**Table 4:** Total count, differences between 2017 and 2002 in percent for lower leg fractures for men and women. Total count for patients > 90 years have been added together

	15–24 years			25–34 years			35–44 years			45–54 years			55–64 years			65–74 years			75–84 years			85–90 years			> 90 years		
	2002	2017	Δ (%)	2002	2017	Δ (%)	2002	2017	Δ (%)	2002	2017	Δ (%)	2002	2017	Δ (%)	2002	2017	Δ (%)	2002	2017	Δ (%)	2002	2017	Δ (%)	2002	2017	Δ (%)
***Male***																											
Proximal Tibia (S82.1)	859	849	−1.2	1077	900	−16.4	2064	1228	−40.5	1989	1968	−1.1	1460	1642	12.5	810	769	−5.1	367	610	66.2	65	101	55.4	25	58	132.0
Tibial Shaft (S82.2)	2172	1374	−36.7	1618	1294	−20	1799	1029	−42.8	1264	1215	−3.9	789	1054	33.6	378	409	8.2	98	270	176.0	9	48	433.3	8	21	162.5
Distal Tibia (S82.3)	1535	878	−42.8	1189	723	−39.2	1990	895	−55.0	1588	1236	−22.2	1136	985	−13.3	562	525	−6.6	161	273	69.6	16	48	200.0	23	31	34.78
Medial Malleolus (S82.5)	841	433	−48.5	574	402	−30	581	365	−37.2	424	420	−0.9	279	354	26.9	147	161	9.5	62	120	93.6	7	13	85.7	4	1	−75.0
Lateral Malleolus (S82.6)	3835	2420	−36.9	3890	2431	−37.5	5187	2174	−58.1	3994	3251	−18.6	3522	2911	−17.4	2591	1946	− 24.9	819	1554	89.7	112	303	170.5	35	80	128.6
Other parts (S82.8)	1339	850	−36.5	1662	1174	−29.3	2332	1232	−47.2	2082	1661	−20.2	1924	1717	−10.8	1126	1206	7.1	420	834	98.6	61	170	178.7	30	49	63.3
**Overall**	**10,581**	**6804**	**−35.7**	**10,010**	**6924**	**−30.8**	**13,953**	**6923**	**−50.4**	**11,341**	**9751**	**−14.0**	**9110**	**8663**	**−4.9**	**5614**	**5016**	**−10.7**	**1927**	**3661**	**90.0**	**270**	**683**	**153.0**	**125**	**240**	**92.0**
***Female***																											
Proximal Tibia (S82.1)	354	350	−1.1	627	613	−2.2	942	694	−26.3	1183	1748	47.8	1980	2886	45.8	2041	2139	4.8	1860	2170	16.7	431	622	44.3	310	432	39.4
Tibial Shaft (S82.2)	389	352	−9.5	456	368	−19.3	717	433	−39.6	711	751	5.6	690	709	2.8	450	503	11.8	345	519	50.4	104	183	76.0	105	159	51.4
Distal Tibia (S82.3)	318	245	−23	448	354	−21	848	490	−42.2	952	845	−11.2	1024	892	−12.9	766	714	−6.8	502	761	51.6	182	237	30.2	125	225	80.0
Medial Malleolus (S82.5)	264	195	−26.1	156	144	−7.7	221	106	−52.0	176	193	9.7	266	264	−0.8	232	225	−3.0	188	237	26.1	41	64	56.1	22	45	104.6
Lateral Malleolus (S82.6)	1307	1069	−18.2	2264	1409	−37.8	4272	1755	−58.9	5082	4113	−19.1	6413	4450	− 30.6	4939	3601	−27.1	2383	3310	38.9	432	679	57.2	203	386	90.2
Other parts (S82.8)	669	586	−12.4	1190	882	−25.9	2493	1145	−54.1	3357	2803	−16.5	5080	4402	−13.4	4357	4178	−4.1	2603	4082	56.8	491	960	95.5	261	582	123.0
**Overall**	**3301**	**2797**	**−15.3**	**5141**	**3770**	**−26.7**	**9493**	**4623**	**−51.3**	**11,461**	**10,453**	**−8.8**	**15,453**	**13,603**	**−12.0**	**12,785**	**11,360**	**−11.1**	**7881**	**11,079**	**40.6**	**1681**	**2745**	**63.3**	**1026**	**1829**	**78.3**

**Fig. 6:**
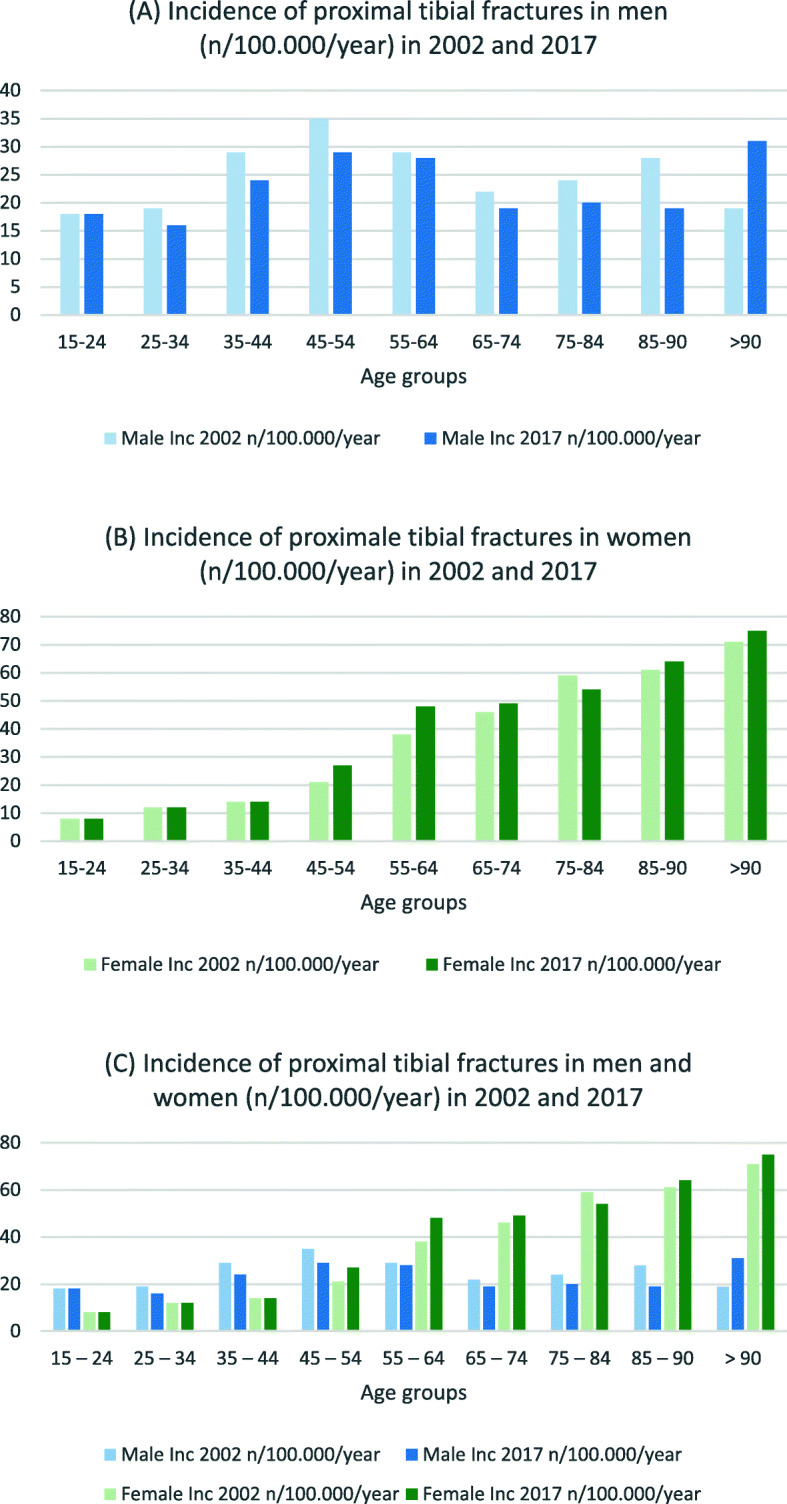
Incidences of proximal tibial fractures in **a** men, **b** women and **c** both (n/100,000/year) for 2002 and 2017

**Fig. 7:**
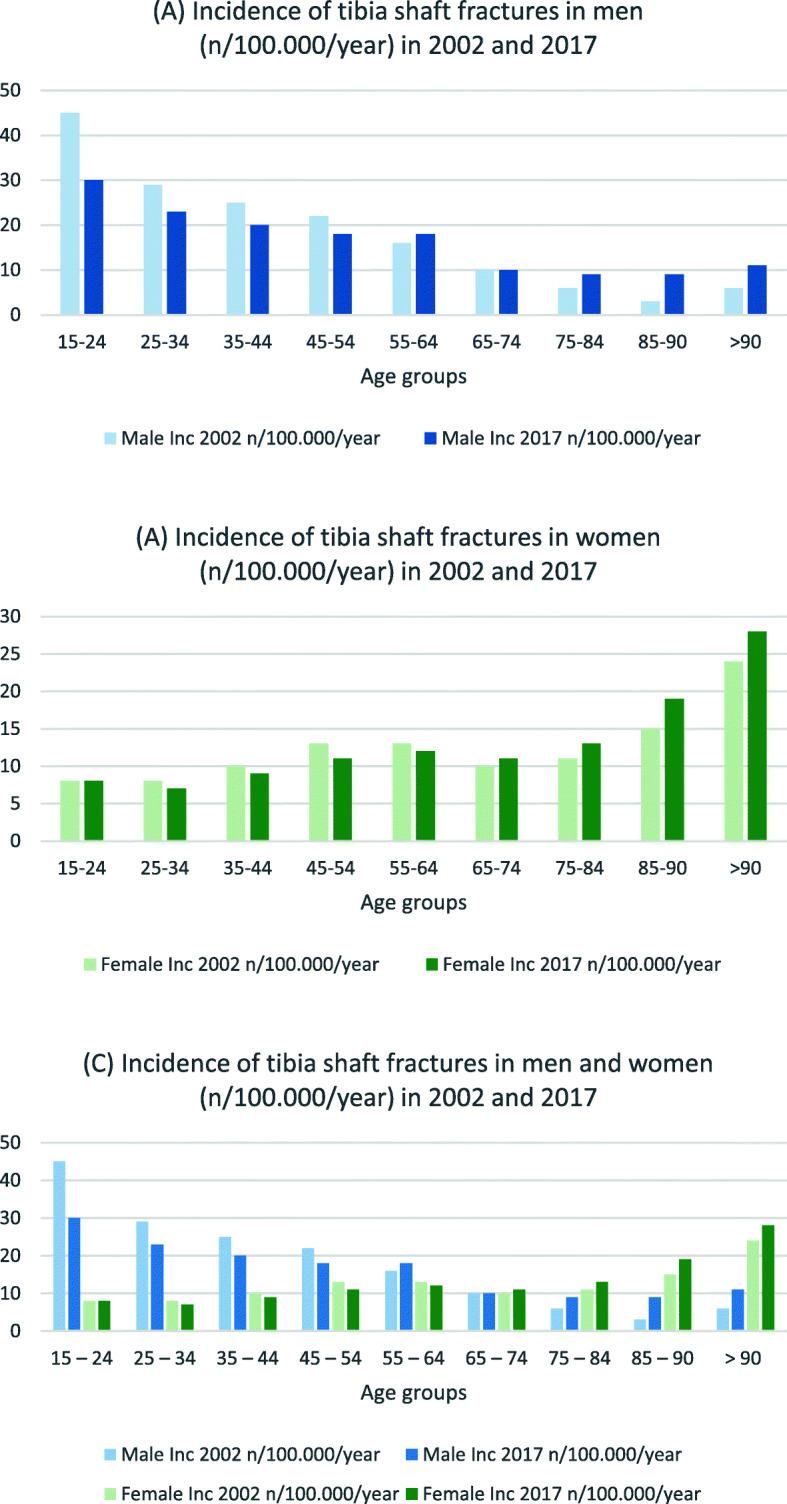
Incidences of tibial shaft fractures in **a** men, **b** women and **c** both (n/100,000/year) for 2002 and 2017

**Fig. 8:**
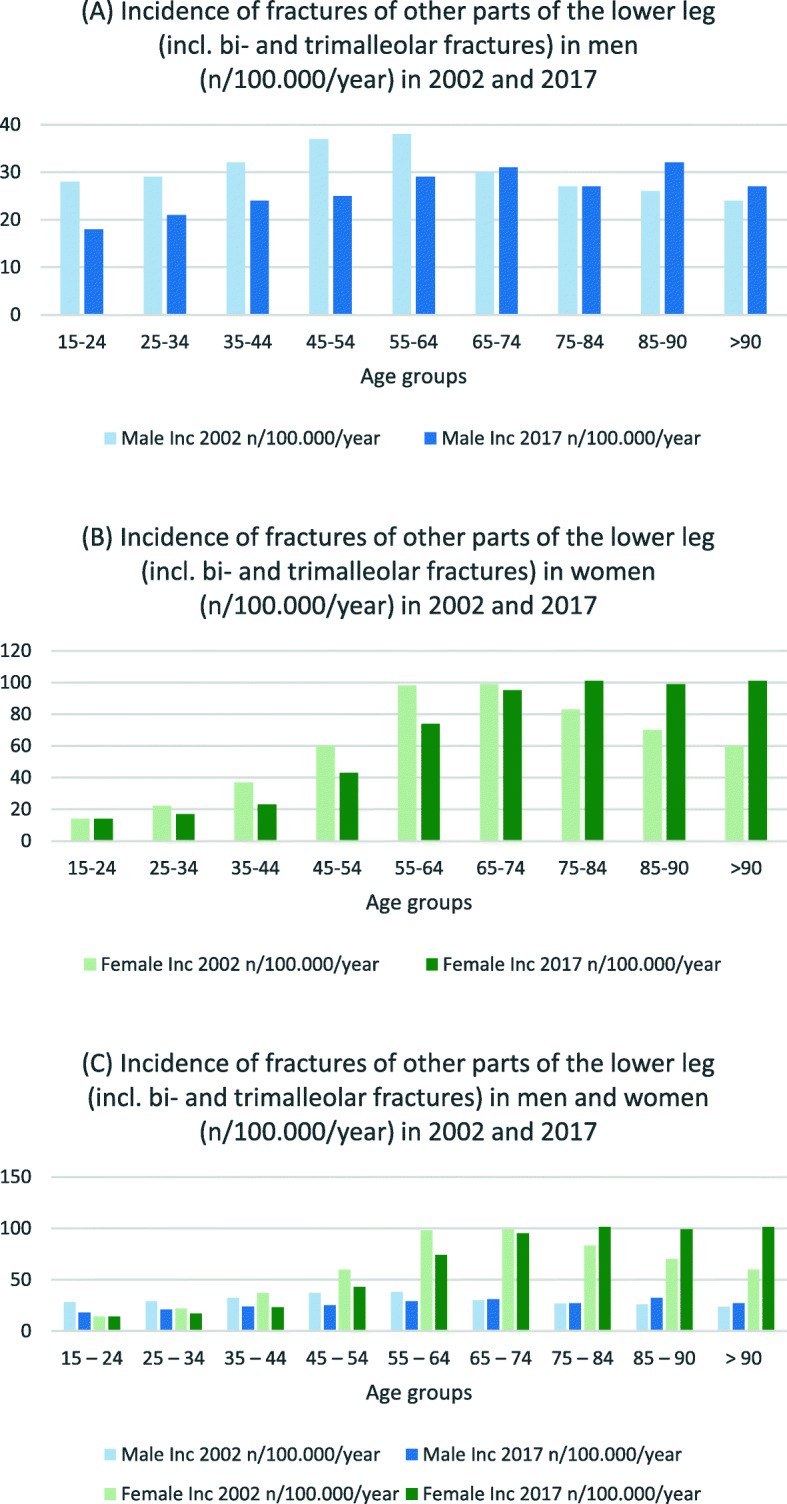
Incidences for fractures of other parts of the lower leg (including bi- and trimalleolar fractures) in **a** men, **b** women and **c** both (n/1000,000/year) for 2002 and 2017

Table 5 presents lower leg fracture incidences for both sexes. For the two youngest male age groups, incidence decreased for nearly fracture type between 2002 and 2017 (IRR 0.5–0.8). Incidences of male proximal tibial fractures decreased in seven of nine age groups between these 3 years (see Fig. 6), whereas tibial shaft fractures increased for those age 55 years and older (IRR 1.0–1.8) (see Fig. 7). Proximal tibial fracture incidence in women increased starting at the age of 45 years (IRR 1.1–1.3) except for the age group 75–84 years (IRR 0.9). Tibial shaft fracture incidence increased starting at age 75 years (IRR 1.2–1.3). Fractures of other parts of the lower leg (including bi- and trimalleolar fractures) were seen in women age 65 years and older at three to four times the incidence rate for the corresponding male cohort (see Fig. 8).

**Table 5:** Incidences of lower leg fractures in 2002 and 2017 by gender (n/100,000/year), IRR: Incidence rate ratio

	15–24 years			25–34 years			35–44 years			45–54 years			55–64 years			65–74 years			75–84 years			85–90 years			> 90 years		
	2002	2017	IRR	2002	2017	IRR	2002	2017	IRR	2002	2017	IRR	2002	2017	IRR	2002	2017	IRR	2002	2017	IRR	2002	2017	IRR	2002	2017	IRR
***Male***																											
Proximal Tibia (S82.1)	18	18	1.0	19	16	0.8	29	24	0.8	35	29	0.8	29	28	1.0	22	19	0.9	24	20	0.8	28	19	0.7	19	31	1.6
Tibial Shaft (S82.2)	45	30	0.7	29	23	0.8	25	20	0.8	22	18	0.8	16	18	1.1	10	10	1.0	6	9	1.5	3	9	3.0	6	11	1.8
Distal Tibia (S82.3)	32	19	0.6	21	13	0.6	28	17	0.6	28	18	0.6	22	17	0.8	15	13	0.9	10	9	0.9	7	9	1.3	18	17	0.9
Medial Malleolus (S82.5)	17	9	0.5	10	7	0.7	8	7	0.9	7	6	0.9	5	6	1.2	4	4	1.0	4	4	1.0	3	2	0.7	3	1	0.3
Lateral Malleolus (S82.6)	79	53	0.7	69	44	0.6	72	43	0.6	71	49	0.7	70	50	0.7	69	50	0.7	53	51	1.0	48	58	1.2	28	43	1.5
Other parts (S82.8)	28	18	0.6	29	21	0.7	32	24	0.8	37	25	0.7	38	29	0.8	30	31	1.0	27	27	1.0	26	32	1.2	24	27	1.1
***Female***																											
Proximal Tibia (S82.1)	8	8	1.0	12	12	1.0	14	14	1.0	21	27	1.3	38	48	1.3	46	49	1.1	59	54	0.9	61	64	1.0	71	75	1.1
Tibial Shaft (S82.2)	8	8	1.0	8	7	0.9	10	9	0.9	13	11	0.8	13	12	0.9	10	11	1.1	11	13	1.2	15	19	1.3	24	28	1.2
Distal Tibia (S82.3)	7	6	0.9	8	7	0.9	12	10	0.8	17	13	0.8	20	15	0.8	17	16	0.9	16	19	1.2	26	24	0.9	29	39	1.3
Medial Malleolus (S82.5)	6	5	0.8	3	3	1.0	3	2	0.7	3	3	1.0	5	4	0.8	5	5	1.0	6	6	1.0	6	7	1.2	5	8	1.6
Lateral Malleolus (S82.6)	28	26	0.9	42	27	0.6	63	35	0.6	91	63	0.7	124	75	0.6	113	82	0.7	76	82	1.1	62	70	1.1	46	67	1.5
Other parts (S82.8)	14	14	1.0	22	17	0.8	37	23	0.6	60	43	0.7	98	74	0.8	99	95	1.0	83	101	1.2	70	99	1.4	60	101	1.7

In 2002, 24,037 ft fractures were registered. In 2017 there was a decrease to 22,519 (− 6.3%). 15,602 fractures occurred in men (65%) in 2002 and 8435 in women (35%). In 2017, 12,910 (57.3%) fractures could be detected for men and 9606 (42.7%) for women (− 17.3 and 13.9% respectively).

Table 6 presents total counts of foot fractures in the years 2002 and 2017 together with percentage change. There was a decrease of absolute numbers in the three youngest age groups. At the same time the fracture rate in the three oldest male age groups increased for nearly all fractures. In women the rate of foot fractures increased in all age groups except age group 15–24 and 35–44 years.

**Table 6:** Total count, differences between 2017 and 2002 in percent for fractures of foot and toe (except ankle) for men and women. Total count for patients > 90 years have been added together

	15–24 years			25–34 years			35–44 years			45–54 years			55–64 years			65–74 years			75–84 years			85–90 years			> 90 years		
	2002	2017	Δ (%)	2002	2017	Δ (%)	2002	2017	Δ (%)	2002	2017	Δ (%)	2002	2017	Δ (%)	2002	2017	Δ (%)	2002	2017	Δ (%)	2002	2017	Δ (%)	2002	2017	Δ (%)
***Male***																											
Calcaneus (S92.0)	403	213	−47.1	882	584	−33.8	1840	853	−53.6	1683	1278	−24.1	1127	1060	− 5.9	468	373	−20.3	92	146	58.7	14	24	71.4	3	3	0
Talus (S92.1)	336	229	−31.8	337	259	−23.1	312	155	−50.3	151	188	24.5	93	97	4.3	31	38	22.6	7	10	42.9	0	2	–	0	0	0
Other and unspecific tarsal bone(s) (S92.2)	145	141	−2.8	138	159	15.2	166	124	−25.3	120	129	7.5	59	137	132.2	17	23	35.3	10	22	120	1	4	300	2	2	0
Metatarsal bone(s) (S92.3)	1070	889	−16.9	972	995	2.4	1295	866	−33.1	932	1070	14.8	635	882	38.9	325	326	0.3	124	238	91.9	20	41	105	6	22	266.7
Great toe and other toes (S92.4 + S92.5)	384	190	−50.5	228	205	−10.1	333	174	−47.7	200	255	27.5	138	221	60.1	73	118	61.6	28	82	192.9	7	11	57.1	2	4	100
Other parts (S92.7 + S92.9)	84	13	−84.5	70	12	−82.9	95	6	−93.7	61	17	−72.1	59	13	−78	18	3	−83.3	4	3	−25	2	1	−50	0	0	0
**Overall**	**2422**	**1675**	**−30.8**	**2627**	**2214**	**−15.7**	**4041**	**2178**	**−46.1**	**3147**	**2937**	**−6.7**	**2111**	**2410**	**14.2**	**932**	**881**	**−5.5**	**265**	**501**	**89.1**	**44**	**83**	**88.6**	**13**	**31**	**138,5**
***Female***																											
Calcaneus (S92.0)	105	74	−29.5	162	143	−11.7	247	176	−28.7	322	284	−11.8	406	441	8.6	354	353	−0.3	311	334	7.4	55	76	38.2	27	39	44.4
Talus (S92.1)	166	126	−24.1	71	91	28.2	62	56	−9.7	40	63	57.5	39	54	38.5	37	36	−2.7	21	33	57.1	5	9	80	2	5	150
Other and unspecific tarsal bone(s) (S92.2)	62	62	0	47	90	91.5	74	66	−10.8	81	98	21	74	104	40.5	42	66	57.1	51	46	−9.8	10	11	10	3	3	0
Metatarsal bone(s) (S92.3)	387	442	14.2	436	566	29.8	747	614	−17.8	750	1065	42	1004	1333	32.8	800	907	13.4	608	908	49.3	143	172	20.3	59	105	78
Great toe and other toes (S92.4 + S92.5)	88	54	−38.6	51	71	39.2	79	54	−31.6	65	92	41.5	48	72	50	42	49	16.7	54	69	27.8	11	29	163.6	8	24	200
Other parts (S92.7 + S92.9)	22	2	−91	14	1	−92.9	30	5	−83.3	25	11	−56	24	8	−66.7	31	7	−77.4	21	8	−61.9	6	2	−66.7	6	0	–
**Overall**	**830**	**760**	**− 8.4**	**781**	**962**	**23.2**	**1239**	**971**	**−21.6**	**1283**	**1613**	**25.7**	**1595**	**2012**	**26.1**	**1306**	**1418**	**8.6**	**1066**	**1398**	**31.1**	**230**	**299**	**30**	**105**	**176**	**67.6**

Table 7 presents the incidences and IRR of each foot fracture location for males and females in 2002 and 2017. Hereby, fractures incidences of calcaneus and talus fractures decreased in all age groups or remained at the same level in 2017 compared to 2002 (IRR ≤1.0).

**Table 7:** Incidences of fractures of foot and toe (except ankle) in 2002 and 2017 by gender (n/100,000/year), IRR: Incidence rate ratio

	15–24 years			25–34 years			35–44 years			45–54 years			55–64 years			65–74 years			75–84 years			85–90 years			> 90 years		
	2002	2017	IRR	2002	2017	IRR	2002	2017	IRR	2002	2017	IRR	2002	2017	IRR	2002	2017	IRR	2002	2017	IRR	2002	2017	IRR	2002	2017	IRR
***Male***																											
Calcaneus (S92.0)	16	11	0.7	16	10	0.6	25	16	0.6	30	19	0.6	22	18	0.8	12	10	0.8	6	5	0.8	6	5	0.8	2	2	1.0
Talus (S92.1)	3	2	0.7	6	5	0.8	4	3	0.8	3	3	1.0	2	2	1.0	1	1	1.0	0	0	–	–	0	–	–	–	–
Other and unspecific tarsal bone(s) (S92.2)	2	2	1.0	2	3	1.5	2	2	1.0	2	2	1.0	1	2	2.0	0	1	–	1	1	1.0	0	1	–	2	1	0.5
Metatarsal bone(s) (S92.3)	14	14	1.0	17	18	1.1	18	17	0.9	16	16	1.0	13	15	1.2	9	8	0.9	8	8	1.0	9	8	0.9	5	12	2.4
Greater toe (S92.4)	2	2	1.0	3	3	1.0	3	2	0.7	2	3	1.5	2	3	1.5	1	2	2.0	1	2	2.0	2	2	1.0	–	2	–
Lesser toe(s) (S92.5)	1	1	1.0	2	1	0.5	2	1	0.5	1	1	1.0	1	1	1.0	1	1	1.0	1	1	1.0	1	0	0.0	2	1	0.5
Other fractures of foot, except ankle (S92.7)	1	0	0.0	1	0	0.0	1	0	0.0	1	0	0.0	1	0	0.0	0	0	–	0	0	–	0	–	–	–	–	–
Unspecific fractures of foot and toe (S92.9)	0	0	–	0	0	–	1	–	–	1	0	0.0	0	0	–	0	0	–	0	0	–	0	0	–	–	–	–
***Female***																											
Calcaneus (S92.0)	5	5	1.0	3	3	1.0	4	3	0.8	6	4	0.7	8	7	0.9	8	8	1.0	9	8	0.9	8	8	1.0	6	7	1.2
Talus (S92.1)	1	1	1.0	1	2	2.0	1	1	1.0	1	1	1.0	1	1	1.0	1	1	1.0	1	1	1.0	1	1	1.0	0	1	–
Other and unspecific tarsal bone(s) (S92.2)	1	1	1.0	1	2	2.0	1	1	1.0	1	2	2.0	1	2	2.0	1	2	2.0	2	1	0.5	1	1	1.0	1	1	1.0
Metatarsal bone(s) (S92.3)	12	15	1.3	8	11	1.4	11	12	1.1	14	16	1.1	19	23	1.2	18	21	1.2	19	21	1.1	20	18	0.9	13	18	1.4
Greater toe (S92.4)	1	1	1.0	1	1	1.0	1	1	1.0	1	1	1.0	0	1	–	0	1	–	1	1	1.0	1	2	2.0	1	2	2.0
Lesser toe(s) (S92.5)	1	1	1.0	0	0	–	1	0	0.0	1	1	1.0	0	0	–	0	0	–	1	1	1.0	1	1	1.0	0	2	–
Other fractures of foot, except ankle (S92.7)	0	0	–	0	–	–	0	0	–	0	0	–	0	0	–	0	0	–	0	0	–	0	0	–	0	–	–
Unspecific fractures of foot and toe (S92.9)	0	0	–	0	0	–	0	0	–	0	0	–	0	0	–	0	–	–	1	0	0.0	0	0	–	1	–	–

## Discussion

The goal of this study was to analyse changes in the counts and incidence rates of the various fractures of the lower extremities for both sexes over a period of 15 years.

It is a well-known fact that geriatric population (> 65 years) is increasing and the fracture incidence in that group is also increasing. But so far there are few studies that provide a dedicated overview of all fractures of the lower extremity.

In this analysis, hip and femur fractures increased by 23.5% in 2017 compared to 2002. Women still suffer more fractures than men, but the proportion occurring in men increased from 26.8% in 2002 to 31.7% in 2017. There has been a notable decrease for both sexes in the total number of fractures in the lower leg by − 15.4%, with most of these occurring in women: 52% in 2002 and 56.1% in 2017.One main reason seems to be the demographic change, with a decrease in the size of younger population groups. The absolute population of 25- to 65-year-olds was 68.3 million in 2002 and decreased to 65.0 million in 2017 (− 5%). By contrast, the absolute number of people over 65 years of age grew from 14.1 million in 2002 to 17.5 million in 2017, an increase of 24% [27].

Age groups older than 45 years shows an increasing population. For example, the age group 15–24 years decreased in total from 9,514,459 in 2002 to 8,683,081 in 2017 (− 8,7%), the age group of 75–84 years increased from 4,769,704 in 2002 to 7,120,634 in 2017 (49.3%) [28]. The ageing population grows and with it the concomitant increase of total fracture counts and incidences. Other possible explanations for the decrease in fractures within the younger groups are the increases in road safety and occupational safety. Accidents involving men and personal injuries decreased by 10% over the same time period [29]. In 2017 there were 26% fewer accidents at work than in 2002 [30]. It must be noted, however, that direct evidence of a causal relationship between these factors and the decline of fractures in younger age groups is lacking.

Kannus et al. reported a decline in the incidence of hip fractures among Finnish people > 50 years of age between 1970 and 2016 [20]. They observed, however, that incidence rates will increase by 44% by the year 2030 due to the sharp growth of the population at risk [20]. An analysis of health insurance data on 23 million Germans from 2002 to 2004 demonstrated that the incidence of hip fractures increases with age [31]. The current study confirms this observation. Using data from the Swedish fracture registry, Mattisson et al. found that women accounted for 69% of all hip fractures, demonstrating a distribution by sex for this condition similar to that seen in the current study [32]. In 2017, 69% of all hip and femur fractures in Germany occurred in women.

The incidence of femoral shaft fractures increased steeply starting at the age of 75 years. Weiss et al. reported the same results, although they found that the incidence remained stable between 1998 and 2004 [33]. They reported that up to the age of 40–49 years, men are more frequently affected by femoral shaft fractures than are women of the same age. This sex difference changed for those 60–69 years and older, with an incident rate ratio for men to women of 0.7 to 0.3 for those over the age of 90 years [33]. The current data confirm this finding. Court-Brown et al. investigated 4786 fractures in inpatients and outpatients over two one-year periods (July 2007–June 2008 and September 2010–August 2011) in Scotland and analysed incidences of different fracture ‘patterns’ in patients > 65 years of age. He described a correlation between increasing age and increasing incidence of fractures of the proximal femur and femoral shaft in both sexes [2]. Our results are consistent for femoral shaft fractures and per- and subtrochanteric fractures.

The steep increase in the incidence of distal femoral fractures with increasing age in women is noteworthy. Elsoe et al. reported a rapid, continuous increase in incidence after the age of 60 with an increasing proportion of female patients after analysing 293 patients with 302 distal femur fractures between 2005 and 2010 [34]. Court-Brown et al. also detected an increasing incidence of distal femoral fractures for women [2]. It should be discussed whether fractures of the distal femur, which increase in incidence with older age in women, should be seen as fragility fractures [34]. The simultaneous presence of osteoporosis in elderly women supports this assumption.

Women showed an increasing number and incidence of proximal tibial fractures, especially for age groups 55 years and older. Court-Brown et al. also recorded an increase in proximal tibial fractures for women with increasing age [2], and a Swedish registry study from 2018 demonstrated an increasing incidence of such fractures for women with increasing age [18]. Vestergaard et al. showed similar results in their cohort study of over 60,000 patients [19]. A direct comparison of incidence rates in this work with those reported from other countries is not possible due to differences in study designs, data sources, time periods, and statistical methods. Nevertheless, time trends and sex distributions may be comparable.

Tibial shaft fractures increased in incidence for both sexes older than 65 years. Wennergren et al. were able to demonstrate an increase in the incidence of fractures at all tibia locations in women with increasing age based on data from the Swedish fracture registry [18]. Taking into account minor variations, these results are in line with data from the current study. Consideration should be given to whether fractures of the tibia (regardless of which segment) should be seen as fragility fractures due to their high incidence in older women (> 65 years) and the concomitant presence of osteoporosis [18]. In 2008, based on Swedish registry data, Weiss et al. were able to identify an increasing incidence of tibial shaft fractures for women > 70 years of age [35].

Court-Brown et al. described an increasing incidence of ankle fractures in both sexes for those > 65 years of age [36]. Although a Finnish registry study showed a decreasing incidence of ankle fractures between 1970 and 2014 for patients > 60 years of age, the authors pointed out that an increasing number of fractures can be expected due to the rapid ageing of the Finnish population [13]. Available data in this study indicates this assumption is also likely for Germany. A Swedish registry study with more than 91,000 patients showed an increasing incidence of ankle fractures in women over the fifth decade of life [37]. Such an increase is also evident in the present study in the incidence rates for ankle fractures and fractures from the S82.8 group, which includes bi- and trimalleolar fractures. After analysing data gathered between 1987 and 2004 on 91,410 Swedish inpatients, Thur et al. recorded the greatest increase in the incidence of ankle fractures in women over 60 years of age [37]. In the current study the largest increase in the incidence of ankle fractures from 2002 to 2017 was recorded for the female cohort aged 75–85 years.

The increase in incidence rates for almost all fracture types in the older female cohorts (> 75 years) is conspicuous. The number of older people – and thus older women (> 75 years) – is increasing due to higher life expectancy [28]. In 2002 there were almost 5.5 million men and 8.6 million women older than 65 living in Germany. In 2017 the number of men older than 65 was 7.6 million, with 9.9 million women older than 65 [27]. Furthermore, seniors today are more physically active than in the past [38], which predisposes them to a fracture event. This could explain the increasing total count of hip fractures in men. Since women are five times more likely to develop osteoporosis than men, and osteoporosis manifests itself much earlier in women [7], their risk of suffering a fracture is increased. Due to their higher life expectancy and the risk of falling again, women are particularly at risk [39, 40] for further fracture events (re-fractures). It should be remembered, however, that men can also suffer from osteoporosis. The German osteoporosis guideline group recommends basic diagnostics for women and men starting at the age of 70 years due to the increased risk of fractures [41]. Furthermore, the guideline identifies singular non-vertebral fractures after the age of 50 as a moderate risk factor for osteoporotic fractures, independent of bone density and age. However, the guideline excepts ankle fractures as unlikely to be associated with an increased risk of osteoporotic fractures [41]. This guideline recommendation conflicts with our data, which indicate the incidence of ankle fractures increases with age (> 75 years). The data also show an increasing incidence of proximal tibial fractures and distal femoral fractures with age, especially in women. Consideration should be given to characterise these fractures as fragility fractures.

This study has the several limitations. The query of the national hospital discharge diagnosis register includes only inpatients. Outpatients were therefore not included which is the major limitation of this study. This means that the listed total count and incidence rates are potentially too low, and a precise, definitive statement on the frequency distribution of the individual fracture types cannot be made. Nonetheless, fractures of the lower extremity are most often treated in an inpatient setting which means at least one night in the hospital independent of the fracture severity and applied treatment (surgical or conservative). Thus, the fracture incidences of femur and tibia fractures might be approximately accurate. It also indicates a trend towards surgical treatment. This has already been investigated by Court-Brown et al. They were able to show that rates of surgery, especially on the lower extremity, are increasing for patients with higher age [42]. Fractures of the foot might be underestimated in this study due to the outpatient setting. Furthermore, since the registry could only identify fractures based on ICD-10 coding, additional details on the characteristics and severity of the fracture provided by classifications such as AO/OTA coding were not available. Similarly, the data did not indicate which patients had been treated surgically or conservatively. Furthermore, the years 2002 and 2017 do not allow the assumption that fracture numbers were linear change over time.

A particular strength of the present work is the fact that current, Germany-wide data from almost all hospitals are presented. The work also shows developments over the relatively long period of 15 years including all kind of fractures of the lower extremity and might help to better represent the actual medical care situation.

The study demonstrates that demographic change has already influenced the total number and incidence rates of lower extremity fractures. For orthopaedic surgeons, nurses, hospitals and healthcare system fracture care within Germany will change. Despite a decrease in the number of lower leg fractures by 11%, fractures of the hip and femur increased by 26% over the same period. According to absolute numbers of hip fractures, there is a clear increase which is a potential financial problem for hospitals. Aigner et al. were able to show in their study that a deficit of 657 € is incurred per hip fracture treated in a German hospital [3]. The incidence of geriatric lower extremity fractures is rapidly increasing, especially for distal femoral fractures, proximal tibial fractures and ankle fractures. Similar increases are also seen for fractures of the upper extremities [21]. As a result, the health care system will be confronted with an increasing number of geriatric patients, which will pose challenges not only financially but also structurally within hospitals. Multimorbidity and polypharmacy as well as age-appropriate care and subsequent geriatric rehabilitation will become increasingly important. Studies have shown that an interdisciplinary team consisting of physicians (trauma surgeons and geriatric specialists), nurses, physiotherapists and ergotherapists can achieve a better outcome for patients with fragility fractures [43].

## Conclusion

Almost all fracture types in the lower extremities increased for older people aged over 75 years in 2017 compared to 2002. This particularly affects women. In contrast, the youngest age groups showed a decrease for nearly every fracture in the lower leg. This will call for increasing interdisciplinary care within hospitals, and it will present the healthcare system with financial challenges. Therefore, focused prevention programmes should be considered including an extended fracture spectrum in the elderly with distal femoral fractures, proximal tibial fractures, and ankle fractures.

## Data Availability

The datasets analysed in the current study are online publicly available: http://www.gbe-bund.de

## Abbreviations

ICD: International Classification of Diseases
ICD-10-GM: International Classification of Diseases 10th version German modification
RKI: Robert Koch Institute
